# Citywide trauma experience in Mwanza, Tanzania: a need for urgent intervention

**DOI:** 10.1186/1752-2897-7-9

**Published:** 2013-11-11

**Authors:** Phillipo L Chalya, Ramesh M Dass, Mabula D Mchembe, Nkinda Mbelenge, Isdori H Ngayomela, Alphonce B Chandika, Japhet M Gilyoma, Joseph B Mabula

**Affiliations:** 1 Department of Surgery, Catholic University of Health and Allied Sciences-Bugando, Mwanza, Tanzania; 2 Department of Orthopaedic & Traumatology, Catholic University of Health and Allied Sciences-Bugando, Mwanza, Tanzania; 3 Department of Surgery, Muhimbili University of Health and Allied Sciences, Dar Es Salaam, Tanzania

**Keywords:** Trauma, Etiological spectrum, Injury characteristics, Treatment outcome, Tanzania

## Abstract

**Background:**

Trauma remains a leading cause of morbidity and mortality in resource limited countries. There is paucity of published reports on trauma care in Tanzania, particularly the study area. This study was carried out to describe our experiences in trauma management outlining the etiological spectrum, injury characteristics and treatment outcome of trauma patients at our local setting and compare our results with those from other centers in the world.

**Methods:**

A descriptive prospective study of trauma patients was conducted at Bugando Medical Centre from April 2010 to March 2012. Statistical data analysis was done using SPSS software version 17.0.

**Results:**

A total of 5672 trauma patients were enrolled in the study. The male to female ratio was 2.3: 1. The majority of patients were in the 2^nd^ decade of life. Road traffic accident was the most common cause of trauma accounting for 60.7% of cases. The majority of patients (76.6%) sustained blunt injuries. Musculoskeletal (68.5%) and head/neck (52.6%) were the most frequent body region injured. Soft tissue injuries (open wounds) and fractures were the most common injuries accounting for 82.8% and 76.8% respectively. Majority of patients (74.4%) were treated surgically with wound debridement (94.0%) being the most frequently performed procedure. Postoperative complications were recorded in 31.5% of cases.

The overall median duration of hospitalization was 26 days (range 1 day to 144 days). Mortality rate was 16.7%. Patients who had polytrauma, burn injuries and those who had tetanus and long bone fractures stayed longer in the hospital and this was statistically significant (P < 0.001), whereas the age > 65 years, severe trauma, admission Systolic Blood Pressure < 90 mmHg, presence of tetanus, severe head injury, the duration of loss of consciousness, the need for intensive care unit admission and finding of space occupying lesion on CT scan of the brain significantly influenced mortality (P < 0.001).

**Conclusion:**

Trauma resulting from road traffic accidents remains a major public health problem in this part of Tanzania. Urgent preventive measures targeting at reducing the occurrence of road traffic accidents is necessary to reduce the incidence of trauma in this region.

## Background

Trauma continues to be a major public health problem worldwide and it is associated with high morbidity and mortality both in developed and developing countries [1-3]. Trauma is reported to be the leading cause of death, hospitalization, and long-term disabilities in the first four decades of life [2,3]. Globally, traumatic injuries account for nine percent of mortality and 12% of the global burden of diseases in terms of disability-adjusted life years (DALYs) lost and are the third most important cause of overall mortality [3]. According to World Health Organization (WHO), an estimated 5.06 million people die each year as a result of some form of traumatic injuries, comprising almost 9% of all deaths [4]. The WHO has predicted a 40% increase in global deaths owing to injury between 2002 and 2030 [3,4]. In Tanzania, like other developing countries, trauma remains a major public health problem and is a leading cause of morbidity and mortality, especially among previously healthy, productive young people and among males in particular [5,6]. Trauma is the single most common reason for admission to Bugando Medical Centre and is associated with high morbidity and mortality and consumes a significant amount of the health care budget [7].

The causes and pattern of traumatic injuries have been reported in literature to vary from one part of the world to another partly because of variations in infrastructure, civil violence, wars and crime [8]. The causes and pattern of trauma reflect trauma patterns within the community and, as such, can provide a guide to the design of programmes geared toward prevention and treatment [8,9]. The common etiologies of trauma, across the world, are road traffic accidents, falls, assaults, firearm injury, sports and industrial accidents [9].

Road traffic accidents are the commonest cause of blunt injuries in civilian practice accounting for up to 80-90% in some studies and are especially common in teenagers and young adults [9-11]. With increasing use of firearms, arrows and spears the incidence of penetrating traumatic injuries has been increased in civil society [12].

The management of trauma patients poses many challenges to trauma surgeons practicing in resource-limited countries like ours [13]. Trauma care in Tanzania, particularly the study area is limited by the lack of a structured prehospital emergency care program and ineffective ambulance system for transportation of patients from the scene to hospitals. Trauma patients are often brought in by friends, relatives, witnesses or the police. Trauma registries are a useful way of describing patterns of injury in a hospital setting. These registries provide unique demographic and outcome data. Using these data, it is possible to identify trends in injury and, in so doing, design prevention programs and further modify and improve existing programs [14]. Hospital trauma registries are a vital source of information on the pattern of injuries and quality of clinical care. However, such registries are lacking in our environment making documentation of trauma care difficult in this part of Tanzania.

Lack of dedicated trauma centres for caring of trauma patients is also a major problem in our setting as a result majority of trauma patients brought to our hospital are often managed in general surgical wards. Late presentation of the disease coupled with prolonged waiting time at the Accident and Emergency are among the hallmarks of the disease in developing countries including Tanzania.

Despite the burden of the problem in Mwanza City and increased trauma admissions to our centre, traumatic injuries have not received the attention they deserve and the public policy responses to this problem have been muted at regional and national level partly because of lack of local data regarding the problem. This study was conducted in order to provide baseline data to policy makers and other stakeholders who may wish to undertake interventions to improve the quality of trauma care in the country. Since the majority of traumatic injuries are preventable, a clearer understanding of the causes, injury patterns and outcome of these patients is essential for establishment of prevention strategies as well as treatment protocols. The aim of this study therefore, was to describe the causes, injury characteristics and outcome of traumatic injuries in our setting. The study provides basis for establishment of prevention strategies as well as treatment protocols.

## Methods

### Study design and setting

This was a prospective descriptive study involving trauma patients admitted to Bugando Medical Centre (BMC) between April 2010 and March 2012. Bugando Medical Centre is consultant and teaching hospital for the Lake and Western zones of the United Republic of Tanzania. It is a teaching hospital for the Catholic University of Health and Allied Sciences-Bugando (CUHAS-Bugando) and a referral centre for tertiary specialist care for six regions namely Mwanza, Mara, Kagera, Shinyanga, Tabora and Kigoma. BMC is situated along the shores of Lake Victoria in Mwanza City. It has 1000 beds and serves a catchment’s population of approximately 13 million people. There is no trauma centre or established advanced pre-hospital care in Mwanza city as a result all trauma patients are referred to BMC for expertise management. At BMC, trauma patients are triaged (by the triage team) according to injury severity to identify critically injured patients who need rapid surgical intervention or the specialized services. BMC has five operating rooms (OR) with ready access to the OR being available 24 h a day. It has also a 12-bed adult and 10-bed paediatric multi-disciplinary Intensive Care Unit (ICU) which is headed by a consultant anesthesiologist and run by trained ICU nurses. The ICU provides services to all patients (trauma and non-trauma, medical and surgical) requiring advanced airway support, mechanical ventilation, hemodynamic support, and electronic monitoring which are usually not available in the open wards in our hospital. The majority of trauma patients admitted in the ICU come from the Accident and Emergency (A & E) department, operating theatre, wards and others come from other peripheral hospitals.

### Study population

The study population included all trauma patients admitted to BMC during the study period and those who consented for the study. Patients who presented in a “shocked” state and those who were under 18 years of age, their parents, guardian or relatives had to consent on their behalf. Patients who failed to give proper information and those who had no relative to consent for the study were excluded from the study. Patients who were readmitted after discharge were also excluded fro the study.

Recruitment of patients to participate in the study was done at the Accident & Emergency (A & E) department. Patients were screened for inclusion criteria and those who met the inclusion criteria were, after informed consent to participate in the study, consecutively enrolled into the study.

All study patients were first resuscitated in the A & E department (by the admitting surgical team) according to Advanced Trauma Life Support. From the A & E department patients were taken into the surgical wards or the intensive care unit (ICU) from where necessary investigations were completed and further treatment was instituted. Routine investigations including hematological (hemoglobin, blood grouping & cross-matching), biochemical (serum creatinine & serum electrolytes) and radiological (x-rays of the chest & abdomen, abdominal ultrasound and CT scan) were performed on admission.

The severity of injury was determined using the Kampala trauma score II (KTS II) [15] and Glasgow Coma Scale (GCS). According to KTS II, severe injury consisted of a KTS II ≤ 6, moderate injury 7–8, and mild injury 9–10, and according to GCS, patients with head injuries were classified into: severe (GCS 3–8), moderate (GCS 9–12) and mild (GCS 13–15). An initial systolic blood pressure (SBP) on each patient was also recorded on admission. Data were recorded using a questionnaire. Data administered in the questionnaire included details of demographic profile, causes of injury, injury characteristics, trauma scores (KTS II & GCS), treatment offered, complications, length of hospital stay (LOS), mortality and patient disposal. Depending on the type of injury, the patients were treated either conservatively or by surgery. All patients were followed up till discharged or death. Patients were also followed up for 3–6 months after discharge.

### Statistical data analysis

Statistical data analysis was done using SPSS software version 17.0. Data was summarized in form of proportions and frequent tables for categorical variables. Continuous variables were summarized using means, median, mode and standard deviation. P-values were computed for categorical variables using Chi-square (χ^2^) test and Fisher’s exact test depending on the size of the data set. Independent student t-test was used for continuous variables. Multivariate logistic regression analysis was used to determine predictor variables that are associated with outcome. A p-value of less than 0.05 was considered to constitute a statistically significant difference.

### Ethical considerations

The study was carried out after the approval by the department of surgery and BMC/CUHAS-Bugando ethics review board. An informed written consent was sought from patients or relatives.

## Results

### Socio-demographic data

During the period under study, a total of 5672 trauma patients were enrolled in the study. There were 3952 (69.7%) males and 1720(30.3%) females with a male to female ratio of 2.3: 1. The age ranged from 2 months to 76 years with a median age of 28 years. The modal age group was 21–30 years accounting for 52.6% of cases. The majority of patients, 4211(74.2%) had primary or no formal education and most of them (3998, 70.4%) were self-employed (businessmen).

### Circumstances of injury

Road traffic accident was the most common cause of trauma accounting for 60.7% of cases (Table 1). Of these, motorcycle (2120, 61.5%) was responsible for the majority of road traffic accidents, followed by motor-vehicles (1283, 37.2%), bicycle (39, 1.1%) and other means of transport (such as use of animals (e.g. donkey) for transport) in 3 (0.08%) of cases. Pedestrians (1822, 52.9%) accounted for the majority of victims, followed by passengers (943, 27.4%), drivers/riders (674, 19.5%) and others (6, 0.2%). In patients who had motorcycle injuries, helmet use was reported in 453 (21.4%) patients. Other causes of trauma in these patients are shown in Table 1.

**Table 1:** Distribution of patients according to causes of trauma

**Cause of injury**	**Number of patients**	**Percentages**
Road traffic accidents	3445	60.7
Assaults	1074	18.9
Falls	982	17.3
Burns	128	2.3
Animal attack	14	0.2
Self-inflicted	14	0.2
Sport-related	6	0.1
Others	6	0.1
Unknown	3	0.05
**Total**	**5672**	**100**

A total of 3554 (62.7%) injuries were unintentional. Intentional injuries occurred in 2100 (37.0%) patients mainly due to physical violence. The remaining 18 (0.3%) patients were cases of undetermined intent.

Regarding the place of injury, most patients sustained injuries on the road in 3445 (60.7%) patients and at home in 2004 (35.3%) patients. This was followed by recreational 187 (3.3%), working places in 30 (0.5%) and other places in 6 (0.1%) patients.

Most of injuries, 4211 (74.2%) occurred during the day. The vast majority of patients, 4366 (77.0%) reported to the Accident & Emergency department within 24 hours after injury. None of our patients had pre-hospital care.

Regarding the mode of arrival to the hospital, the majority of patients were brought in by relatives and Good Samaritan in 3287 (58.0%) patients. 2360(41.6%) patients were brought in by police and only twenty-five (0.4%) patients were brought in by ambulance. We noted high mortality rates (63.9%) in patients arriving during the night compared with 36.1% of day arrivals. This difference in mortality rates was statistically significant (P < 0.001).

The waiting time (i.e. time interval taken from reception at the Accident & Emergency department and reception of treatment) ranged from 20 minutes to nine hours (median = 3 hours). The majority of patients, 4598(81.1%) were attended to within three hours of arrival to the Accident & Emergency department.

### Injury characteristics

The majority of patients, 4344 (76.6%) sustained blunt injuries and the remaining 1328 (23.4%) had either penetrating injuries or both. Musculoskeletal (Limbs) was the most commonly involved body region in (68.5%) of patients, followed by head & neck in 52.6% of cases (Table 2). Musculoskeletal injuries included open wounds (bruises, laceration, abrasion, contusions, traumatic amputation) in 3218 (82.8%) injuries and fractures in 2986 (76.8%) injuries. Isolated injuries occurred in 4228 (74.5%) patients while 1444 (25.5%) patients sustained multiple injuries. Open wounds such as bruises, laceration, abrasion and contusions were the most common type of injuries accruing in 69.9% of cases (Table 3). According to Kampala Trauma Score II (KTS II), mild (KTS II = 9–10), moderate (KTS II = 7–8) and severe (KTS II ≤ 6) injuries were recorded in 945 (16.7%), 3291 (58.0%) and 1436 (25.3%) patients respectively. The Glasgow coma scale (GCS) in patients with head injuries indicated that most of them, 2114 (70.9%) had moderate to severe injuries. Of the 5672 patients, 3986 (70.3%) had systolic blood pressure (SBP) > 90 mmHg on admission and the remaining 1686 (29.7%) patients had SBP of 90 mmHg and below.

**Table 2:** Distribution of patients according to the body region injured

**Body region injured**	**Frequency**	**Percentage**
Musculoskeletal (Limbs)	3888	68.5
Head/neck	2983	52.6
Abdomen	1329	23.4
Chest	1127	19.9
Genitalia	45	0.8
Pelvis	34	0.6
Spines	12	0.2

**Table 3:** Distribution of patients according to the type of injury

**Type of injury**	**Frequency**	**Percentage**
Open wounds	3967	69.9
Fractures	2214	39.0
Craniocerebral injuries	215	3.8
Visceral injuries	178	3.1
Burns	128	2.3
Hemothorax/pneumothorax/ haemopneumothorax	38	0.7
Other injuries	32	0.6

### Admission and treatment characteristics

The majority of patients, 4866 (85.8%) were admitted in the general/paediatric surgical and orthopaedic wards and the remaining 806 (14.2%) patients who were critically ill were admitted in the intensive care unit (ICU) for ventilatory support. Of the 5672 patients, 4222 (74.4%) were treated surgically of which surgical wound debridement was the most common surgical procedure performed accounting for 94.0% of cases (Table 4).

**Table 4:** Distribution of patients according to surgical procedure performed (N = 4222)

**Surgical procedure performed**	**Frequency**	**Percentage**
Wound debridement	3967	94.0
Surgical treatment of fractures	1644	38.9
Craniotomy/burr holes	112	2.7
Exploratory laparotomy	103	2.4
Skin grafting/flaps	68	1.6
Limb amputation	63	1.5
Underwater seal drainage (pleural tubes)	38	0.9
Other surgical procedures	25	0.6

### Outcome of surgical treatment

Of the 5672 trauma patients, 1786 developed 2314 postoperative complications giving a complication rate of 31.5%. Of these, surgical site infection was the most common postoperative complication accounting for 36.5% of cases (Table 5).

**Table 5:** Distribution of patients according to postoperative complications (N = 2314)

**Postoperative complications**	**Frequency**	**Percentage**
Surgical site infections	844	36.5
Complication of fracture healing	457	19.7
Complications of abdominal surgery	36	1.6
Complications related to limb amputations	19	0.8
Skin grafting/flaps failure	17	0.7
Keloids/hypertrophic scars	15	0.6
Pulmonary infections	12	0.5
Postburn contractures	10	0.4
Tetanus	7	0.3
Cerebral abscess	5	0.2

Note: Tetanus is caused by *Clostridium Tetani*, a gram positive, anaerobic and spore forming bacterium which is found in soil and in animal and human faeces and the usual mode of entry is through open wounds. The diagnosis of tetanus is wholly clinical and based on the presence of one or more of the following:-

1. Trismus.

2. Rigidity of the neck and or abdomen.

3. Reflex spasms.

The overall median duration of hospitalization was 26 days (range 1 day to 144 days). The duration of hospital stay for non-survivors ranged from 1 day to 42 days (median 6 days). The length of ICU stay ranged from 1 day to 46 days (median 5 days). Patients who had polytrauma, burn injuries and those who had tetanus and long bone fractures stayed longer in the hospital and this was statistically significant (P < 0.001).

In this study, a total of 948 patients died giving a mortality rate of 16.7%. According to multivariate logistic regression, the age > 65 years, severe trauma (Kampala Trauma Score II ≤ 6), admission Systolic Blood Pressure < 90 mmHg, presence of tetanus, severe head injury (Glasgow Coma Score = 3–8), the duration of loss of consciousness, the need for ICU admission and finding of space occupying lesion in CT scan brain significantly influenced mortality (P < 0.001).

### Disposal of patients at discharge

Of the 4724 survivors, 4476 (94.8%) had good recovery and were discharged well. One hundred and twenty five (2.6%) were discharged with permanent disabilities. Fifty-six (1.2%) patients were absconded, seventy-four (1.6%) were discharged against medical advice.

### Follow up of patients

Out of 4724 survivors, 2241 (47.4%) patients were available for follow up at three months after discharge and the remaining 2483 (52.6%) patients were lost to follow up.

## Discussion

Globally, trauma remains an important public health problem and contributes significantly to high morbidity, mortality and long term disability [1-3]. In this review, the majority of our patients were young adults in their most reproductive and productive years and showed a male preponderance which is in keeping with findings from other studies [5,16-18]. High incidence of patients in this age group may be attributed to the fact that males in this age group are more mobile with active participation in high risk taking activities. The fact that the economically productive age-group were mostly involved demands an urgent public policy response. Identification of risk taking behavior among trauma patients has potential significance for the prevention of injuries.

Road traffic accidents have been reported to be the commonest cause of blunt injuries in most studies as supported by the present study [11,18]. In this study, the majority of road traffic accidents were due to motorcycle accidents, an emerging popular mode of commercial transportation in Mwanza City as reported previously by Chalya *et al.*[19]. In keeping with other studies [20-23], pedestrians accounted for the majority of road traffic injured patients in our study, but at variant with other local studies which reported passengers as the majority of cases [5,19]. High incidence of pedestrians in this study reflecting low public awareness on road use and therefore pedestrians are less likely to use walking pavements even if they are available. In addition, the absence of pedestrian walkways in most of the roads in developing countries like Tanzania has increased the vulnerability of pedestrians to all motorized vehicles. Findings from this study calls for urgent interventions targeting at reducing the occurrence of road traffic accidents and subsequently reduce the incidence of these injuries in this region.

The finding that most of injuries in the present study occurred during the day agrees with other findings reported elsewhere [19,20]. Increased rate of injuries during the day can be explained by increased traffic jams as well as increased human activities in the city during the day time. Knowing the time of injury in trauma patient is important for prevention strategies and has an impact on the outcome. We also noted that most patients who arrived during night hours had significantly higher mortality rates compared to day arrivals. This can be explained by the fact that during night hours the majority of the senior surgical and auxiliary staff, whom we found to be pivotal in the diagnosis and management of traumatic injuries, were unlikely to be present unless called for difficult cases. In our resource-limited setting, where staff shortage is a challenging problem, re-distribution of the few staff available needs to be designed to address the problem.

The management of trauma patients has several important elements: adequate pre-hospital care, rapid transport to a specialized centre, complex in-hospital care and rehabilitation. The prehospital phase plays a vital role in determining the final outcome of treatment when done appropriately and contributes significantly to reducing morbidity and mortality [19]. In the present study, lack of prehospital care in our patients and the mode, by which patients arrive, shows gaps in the referral system and is clear testimony that pre-hospital care is lacking in the region. Lack of advanced pre-hospital care and trauma centres in Mwanza city coupled with ineffective ambulance system for transportation of patients to hospitals are a major challenge in providing care for trauma patients and have contributed significantly to poor outcome of these patients due to delay in definitive management. Lack of dedicated trauma centres for caring of trauma patients is a major problem in our community and the intensive care unit at our hospital is unable to cope up with a large number of trauma patients as a result majority of patients are still admitted and managed in general surgical wards which are not well equipped in managing trauma patients.

Waiting time in emergency departments may be attributable to many factors and may stretch up to three hours before completion of all necessary procedures, even in developed countries [23]. In the present study, more than 80% were attended to within three hours of arrival to the Accident & Emergency department which is at variant with Lambe *et al.*[24] in the USA who reported a lower mean waiting time of 56 minutes. A waiting time of 30 minutes for a general outpatient clinic is considered reasonable but should be even shorter for emergency visits [25].

Most patients in this study sustained blunt injuries, which is comparable with other studies [19,26,27], but at variant with other studies [28] in which penetrating injuries was the most common mechanism of injury. The high incidence of blunt traumatic injuries in this study is explained by the fact that those patients who had blunt traumatic injuries were mostly involved in road traffic accidents, a common feature of increased motorization in this environment.

In agreement with previous studies [19,20], the present study found that musculoskeletal and head injuries were the most common body region injured. Our high figure of musculoskeletal injuries affecting mainly the lower limbs is attributable to the large number of pedestrians. Pedestrians are unprotected road users and therefore they are highly exposed to high risk of limb injuries [29]. High incidence of head injuries in the present study reflects low use of motorcycle helmets as seen in our study (21.4%). In the present study, no patient who was helmeted at the time of injury sustained head injury reflecting its importance in prevention of head injuries among motorcycle injury patients. The predominance of head injuries in this study indicates a need for coordinated services for neurotrauma.

Soft tissue injuries (open wounds) and fractures were the most common injuries which is consistent with previous observations from other centers although prevalence varies [10,30].

In the present study, the severity of injury was determined using the Kampala trauma score II (KTS II) whose validity and reliability for use in both adults and children was described elsewhere [15]. This scoring system compares favorably with other trauma scoring systems such as the Revised Trauma Score (RTS) and Injury Severity Score (ISS) [31]. According to Kampala Trauma Score II, the majority of patients were found to have moderate to severe injuries which is in agreement with previous study by Chalya *et al.*[32].

Most of our patients were treated surgically, which is in agreement with other similar studies [19,20,32]. The high incidence of surgical treatment in our study is attributable to the high incidence of patients with moderate to severe injuries the majority of which required surgical intervention.

The presence of complications has an impact on the final outcome of trauma patients. In agreement with other studies [19,32], site surgical infections and complications of fractures healing were the most common complications of treatment of traumatic injuries.

The length of hospital stay (LOS) has been reported to be an important measure of morbidity among trauma patients. Prolonged hospitalization is associated with an unacceptable burden on resources for health and undermines the productive capacity of the population through time lost during hospitalization and disability [8]. Our figure for the overall median LOS in the present study was higher than that reported by others [19,27]. Prolonged LOS in our study is attributable to presence of polytrauma patients, burn injuries and those who had tetanus and long bone fractures which took time to heal as majority of them were treated with either skeletal or skin traction and only few patients were treated with open reduction and internal fixation.

The overall mortality rate in this study was higher than that reported elsewhere [27,33,34]. Factors responsible in our study included advanced patient’s age ( > 65 years old), severe trauma (Kampala Trauma Score II ≤ 6), admission Systolic Blood Pressure < 90 mmHg, presence of tetanus, severe head injury (Glasgow Coma Score = 3–8), the duration of loss of consciousness, the need for ICU admission and finding of space occupying lesion in CT scan brain. Addressing these factors responsible for high mortality in our patients is mandatory to be able to reduce mortality associated with these injuries.

Self discharge by patient against medical advice is a recognized problem in our setting and this is rampant, especially amongst trauma patients [35]. Similarly, poor follow up visits after discharge from hospitals remain a cause for concern. These issues are often the results of poverty, long distance from the hospitals and ignorance and need to be addressed.

Lack of dedicated trauma centres for caring of trauma patients, inadequate ICU spaces, limited diagnostic facilities (e.g. Focused Assessment using Sonography in Trauma, CT scan etc.) discharge against medical advice, and the large number of loss to follow up were the major limitations of this study. However, despite these limitations, the study has provided local data that can be utilized by health care providers to plan for preventive strategies as well as establishment of management guidelines for patients with traumatic injuries. The challenges identified in the management of trauma patients in our setting need to be addressed, in order to deliver optimal trauma care for these patients.

## Conclusion

Trauma is an important public health problem accounting for a substantial proportion of all trauma admissions at Bugando Medical Centre. Road traffic accidents continue to be the major etiological factor for traumatic injuries and the commonly affected victims are young adult males in their productive and reproductive age group. Urgent preventive measures targeting at reducing the occurrence of road traffic accidents is necessary to reduce the incidence of traumatic injuries in this region.

## Competing interests

The authors declared that they have no competing interests.

## Authors’ contributions

PLC conceived the study and did the literature search, coordinated the write-up, editing. RMD, MDM, NM, IHN, ABC and JMG participated in the writing of the manuscript, editing and management if patients. JBM participated in the literature search, writing of the manuscript, data analysis, editing and submission of the article. All the authors read and approved the final manuscript.
